# Fluorescent Microscopy: A Useful and Adjunct Tool in Leprosy Diagnosis: A Cross Sectional Study 

**DOI:** 10.30699/ijp.2024.2023590.3263

**Published:** 2025-01-10

**Authors:** Swati Soni, Vaishali Walke, Dinesh Asati, Anand Maurya, Sramana Mukhopadhyay

**Affiliations:** 1 *Peoples College of Medical Sciences and Research Centre, Bhopal (MP), India*; 2 *Path & Lab Medicine, All India Institute of Medical Science, Bhopal. India*; 3 *Department of Dermatology, LN Medical College, Bhopal. India*; 4 *Microbiology Department, All India Institute of Medical Science, Bhopal. India*

**Keywords:** Auramine-rhodamine, Fite-Faraco, Fluorescent stain, Hansen’s Disease

## Abstract

**Background & Objective:**

Leprosy is a chronic infectious disease caused by Mycobacterium leprae. Fite-Faraco (FF) is the routine staining method used to demonstrate the presence of *Mycobacterium leprae *in tissue sections. Fluorescent microscopy (FM) can help visualize lepra bacilli better. The present study compares two methodologies, fluorescent microscopy, and Fite-Faraco, in detecting Mycobacterium leprae in tissue sections.

**Methods:**

Histopathology of skin biopsies in 60 cases of Hansen's were evaluated with FF stain. The performance of Auramine- Rhodamine Fluroscencent stain was compared with conventional FF staining in identifying Lepra bacilli.

**Results:**

A total of 60 clinically and histopathologically confirmed cases of Hansen’s disease were included in this ambispective study. The cases were sub-classified into various histological categories. Auramine-rhodamine fluorescent staining was performed and examined under a fluorescent microscope with an LED light illuminator. The bacteriological index (BI) was calculated under an oil immersion field for both Fite-Faraco (FF) staining and fluorescent microscopy (FM), graded from zero to six plus according to Ridley’s logarithmic scale. Lepra bacilli were identified in 70% of patients on FF staining, while fluorescent microscopy showed positivity in 80%. The mean BI calculated by FM (2.48) was significantly higher than that by the FF method (2.18), and more multibacillary disease was identified by fluorescent staining compared to FF staining.

**Conclusion:**

It is advantageous to use fluorescent microscopy as an adjunct to conventional Fite-Faraco stain especially in cases where the latter fails to detect lepra bacilli and in a clinically suspected multibacillary disease.

## Introduction

Leprosy is a chronic infectious disease caused by Mycobacterium leprae, which expresses itself in different clinicopathological forms, depending on the immune status of the host (1). Leprosy occurs practically in every corner of the globe; however, in tropical countries like India, it is still one of the major problems of public health importance. This issue can be tackled by early diagnosis and timely treatment (2). The clinical findings are always to be supported by a demonstration of Acid-Fast Bacilli (AFB) in combination with histopathological findings, which is a standard practice for leprosy diagnosis (3). Fite-Faraco (FF) is the routinely used staining method to demonstrate *Mycobacterium leprae *in tissue sections (4). The density of bacilli should be about 1000 per cubic millimeter of the tissue to pick a single bacillus in the tissue section. The laborious search for lepra bacilli is tiresome, leading to observer fatigue with chances of false negativity, under-diagnosis, and possible under grading of the disease. Several studies have emphasized the role of fluorescent microscopy as an alternative in this direction, particularly for disease diagnosis, as mycolic acid, a component of the cell wall of *M. leprae*, can be stained with fluorescent dyes and examined under a fluorescent microscope to facilitate the laboratory diagnosis of leprosy (5-7). The present study is undertaken to compare the sensitivity of the two methodologies; fluorescent microscopy and Fite-Faraco in detecting Mycobacterium leprae in tissue sections.

## Material and Methods

This retrospective cross-sectional study was conducted in the Department of Pathology and Lab Medicine, All India Institute of Medical Sciences, Bhopal. The study group comprised 60 clinically diagnosed cases of Hansen’s disease. After informed consent, the skin biopsy was obtained by a dermatologist in the dermatology OPD at AIIMS, Bhopal. Skin punch biopsy specimens received for histopathology over 4 months (January 2021 to April 2021) were included in the present study. Formalin-fixed paraffin-embedded sections were examined for light microscopy after Hematoxylin and Eosin (H & E) stain. Thereafter, these cases were classified according to Ridley and Jopling classification (8,9) into histologic categories as Indeterminate (IL), Tuberculoid (TL), Borderline- Tuberculoid (BT), Mid-Borderline (BB), Borderline Lepromatous (BL) and Lepromatous Leprosy (LL). Fite-Faraco stain to identify Lepra bacilli was also performed and recorded. Auramine-rhodamine fluorescent stain, as recommended by Kuper and May (10), was applied to tissue sections taken on clean, scratch-free glass slides. Egg albumin or any other adhesive was not used to avoid artefactual staining. These sections were then stained with fluorescent dye (auramine-rhodamine) and examined under a fluorescent microscope with an LED light illuminator. Bright yellow fluorescent solid rods were counted as bacilli for the bacteriological index, while all bacillary fragments were excluded.

The bacteriological index (BI) was calculated under an oil immersion field according to Ridley’s logarithmic scale and was graded from zero to six plus, based on the number of bacilli observed in an average microscopic field under a 100× objective. Since BI is a continuous variable, the investigators divided the cases into two groups: BI < 3 and BI > 3, for comparison purposes. Considering Fite-Faraco (FF) as the standard technique, the performance of fluorescent microscopy (FM) was compared with that of the FF staining method.

### Statistical Analysis

Data were tabulated, and statistical analysis was carried out using IBM SPSS Version 22 (SPSS Inc., Chicago, Ill., USA). Pearson’s coefficient (r-value) was applied for correlation between the groups, and the chi-square test was used to determine significance. A P-value < 0.05 was considered statistically significant.

## Results

**Fig. 1:**
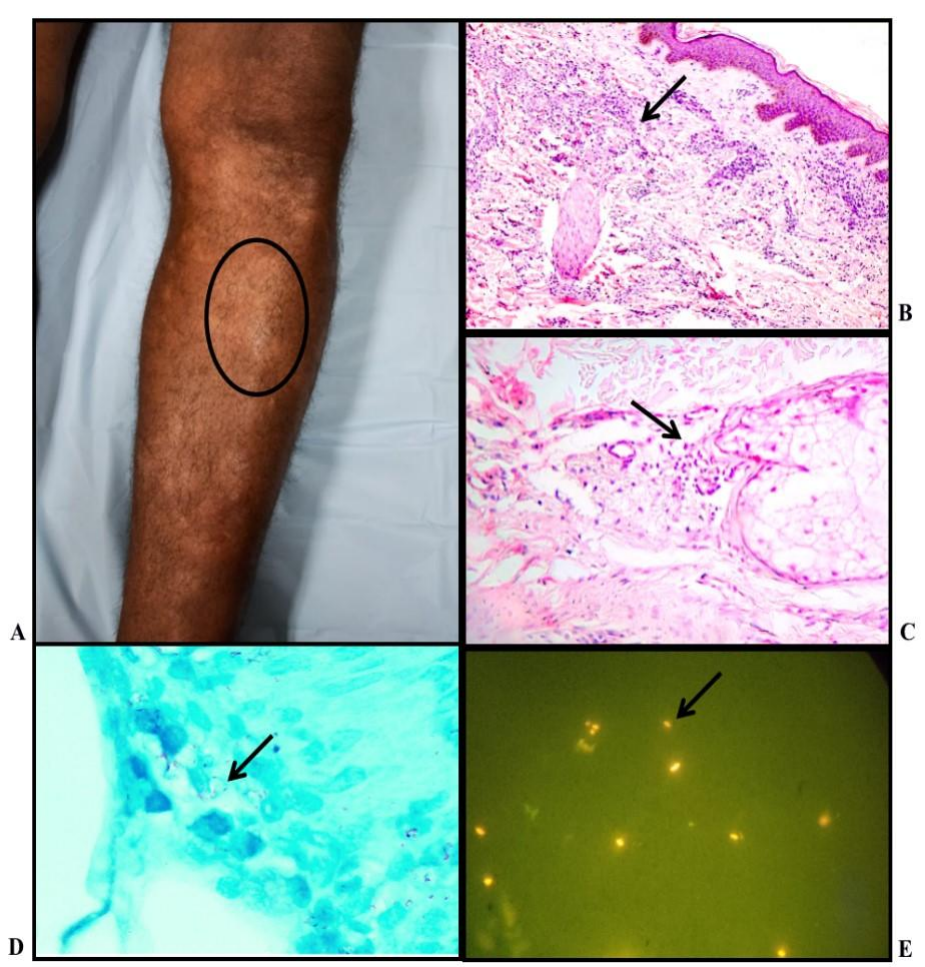
Indeterminate Leprosy: A) Clinical photo: Multiple hypopigmented plaque over leg; B) Histopathology: shows pandermal perineurovascular inflammation (H&E stain, X10); C) Histopathology: shows periadnexal mononuclear inflammation (H&E stain, X20); D) Lepra bacilli seen, bacteriological index - 2+ (FF stain, X100); E) Rod-shaped fluorescent lepra bacilli; BI-2+ (AR stain, X100)

**Fig. 2:**
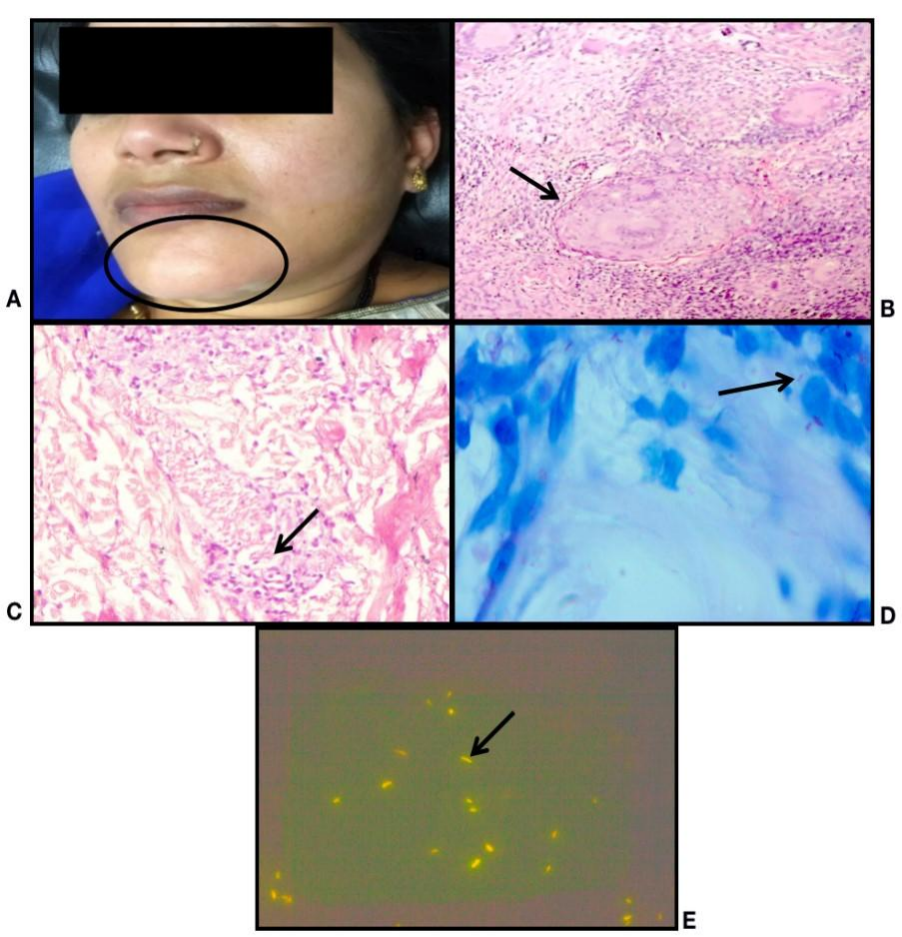
Tuberculoid Leprosy: A) Clinical photo: Erythematous, hypopigmented, barely elevated plaque over left side of chin; B) Histopathology: shows multiple granulomas in the superficial and deep dermis (H&E stain, X10); C) Histopathology: shows perineural mononuclear infiltrate in the deep dermis (H&E stain, X20); D) Lepra bacilli; BI - 2+ (FF Stain, X100); E) Fluorescent lepra bacilli; BI - 3+ (AR stain, X100)

**Fig. 3:**
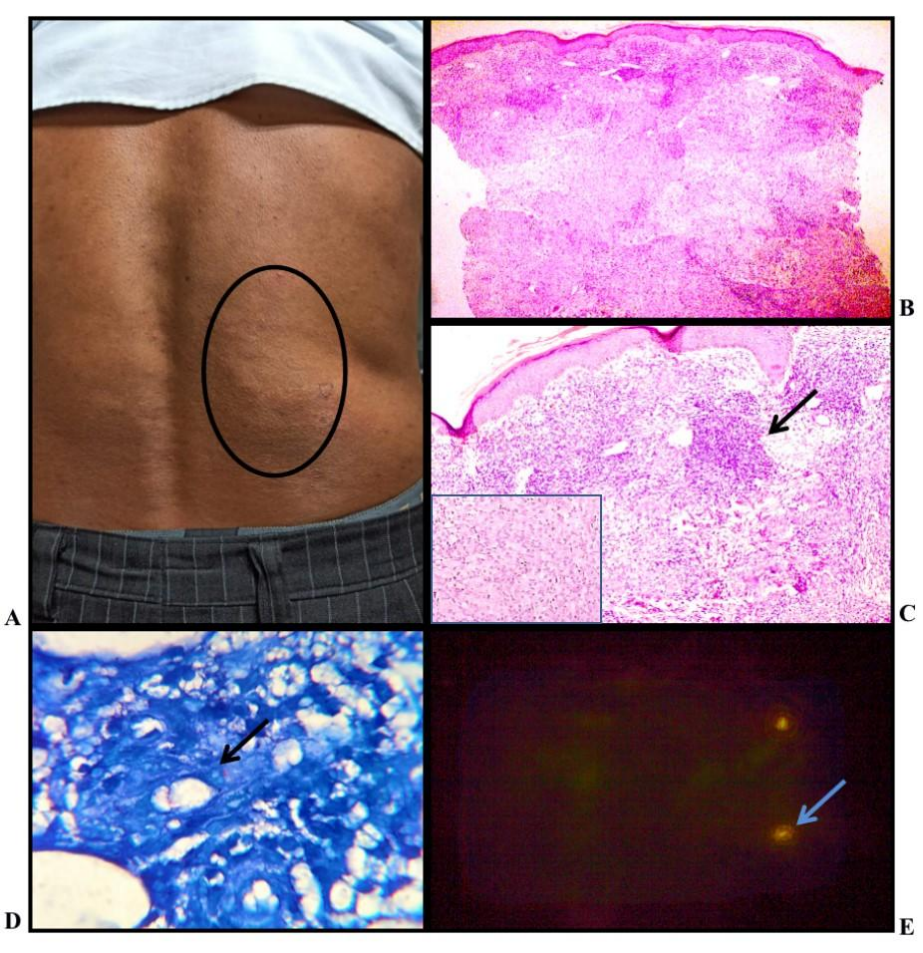
Borderline Tuberculoid Leprosy: (A) Clinical photo : Multiple erythematous annular plaque with satellite lesion present over back; B) Histopathology: shows atrophied epidermis and numerous ill formed granulomas in the superficial and deep dermis (H&E, X4); C) Histopathology: shows flattened epidermis and granuloma present in the superficial dermis, (H&E stain, X10) (Inset: epithelioid histiocytes, X40); D) Occasional lepra bacilli seen, BI - 1+ (FF stain, X100); E) Fluorescent rod shaped bacilli seen; BI - 2+ (AR stain, X100)

**Fig. 4:**
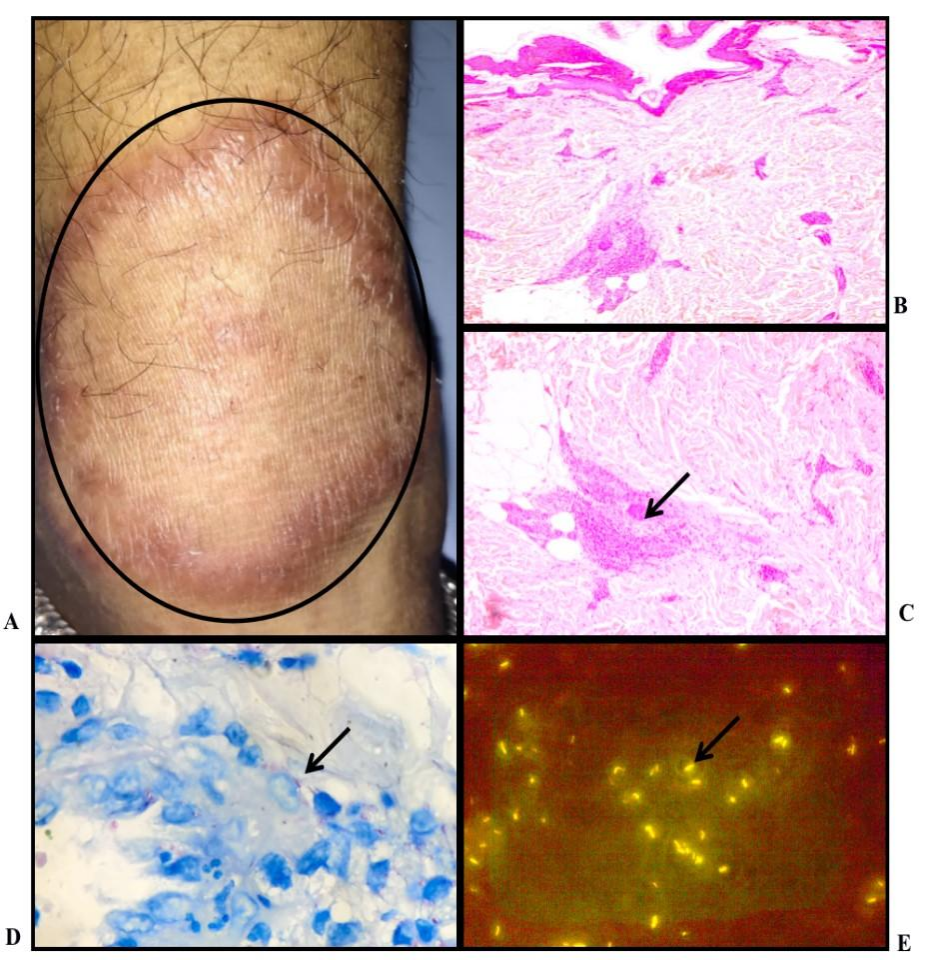
Borderline Lepromatous Leprosy: A) Clinical Photo: Hypopigmented plaque over right cheek; B) Histopathology: Multiple poorly defined granulomas in superficial dermis (H & E stain, X10); C) Histopathology: Perineural predominant lymphocytic infiltrate and collection of few activated macrophages forming ill-defined granuloma seen in the mid dermis (H & E stain, X40); D) Leprae bacilli noted in the cytoplasm of histiocytes and lying free; BI - 5+ (FF stain, X100): E) Large number of leprae bacilli; BI - 5+ (AR stain, X100)

**Fig. 5:**
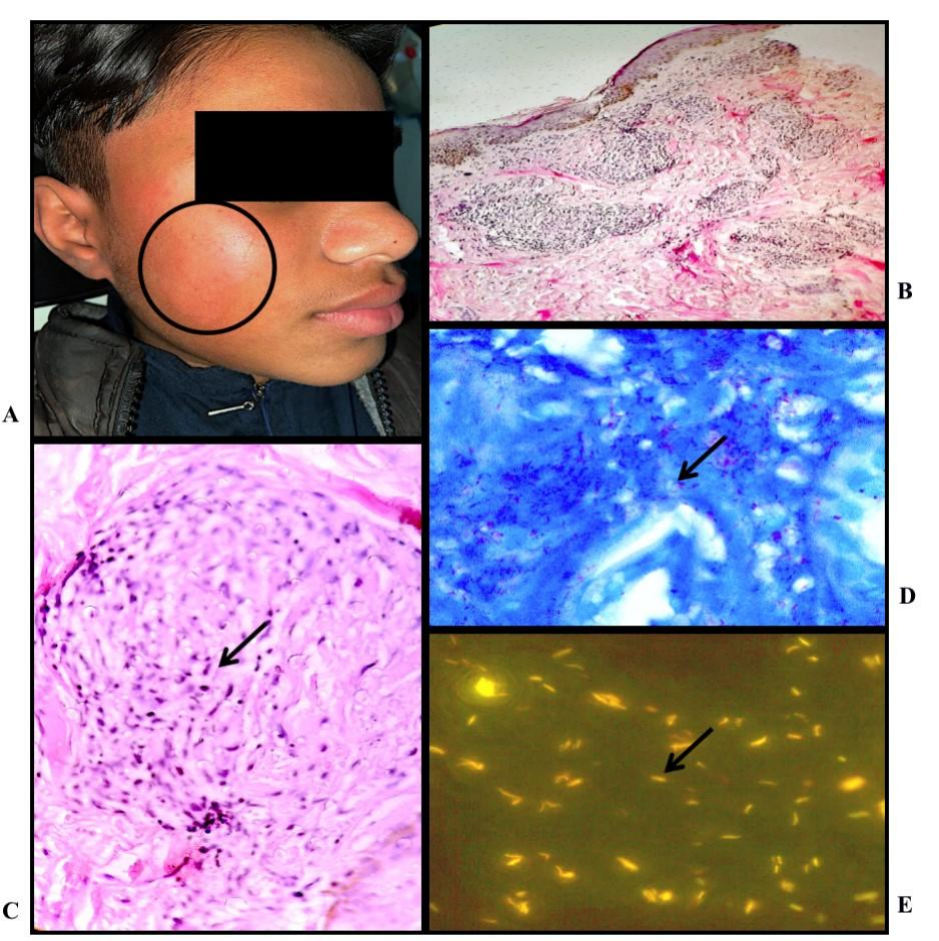
Mid Borderline Leprosy: A) Clinical photo: annular plaque with intact margin and sloping outer margin; B) Histopathology: Atrophied epidermis with Granuloma in mid dermis (H&E stain, X4); C) Histopathology: ill formed granuloma in mid dermis with surrounding peripheral lymphocytes (X10); D) Lepra bacilli within macrophage and, BI - 3+ (FF stain, X100); E) Fluorescent lepra bacilli, BI - 4+ (AR stain, X100)

**Fig. 6:**
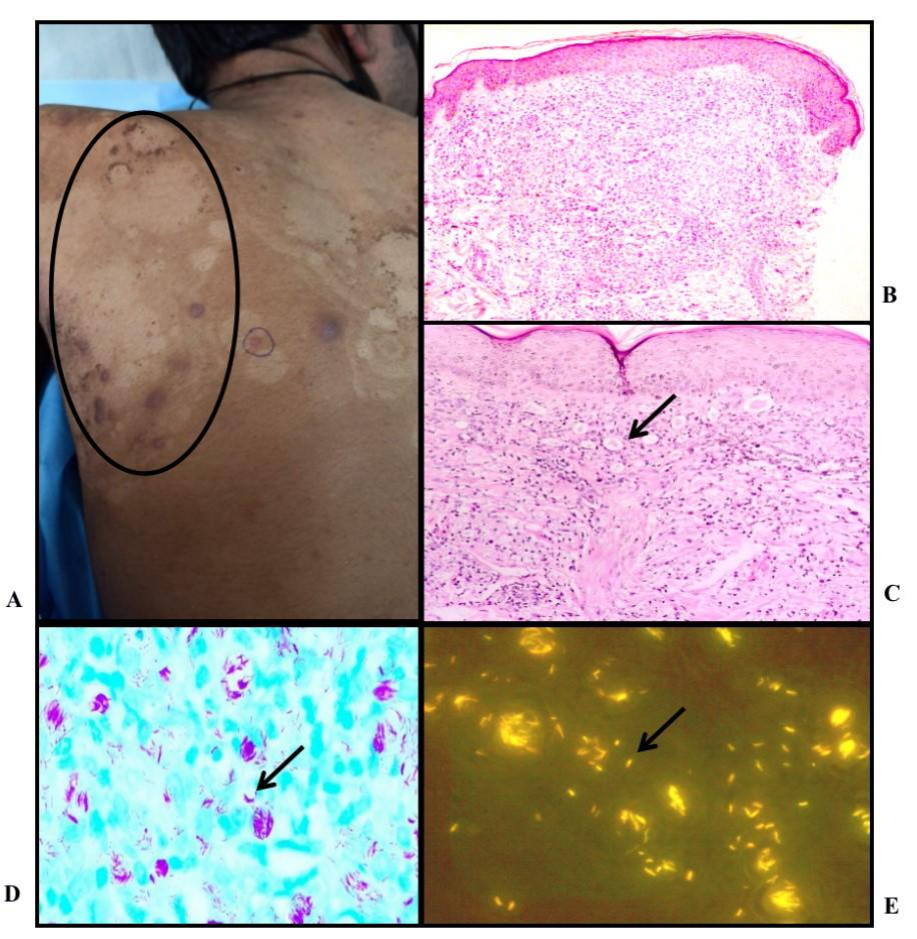
Lepromatous Leprosy: A) Clinical photo: Multiple, ill-defined, hypopigmented macule present over back; B) Histopathology: Pandermal lymphohistiocytic infiltrate leaving a clear grenz zone under the epidermis (H&E;X10); C) Histopathology: foamy macrophages better seen in the superficial dermis (H&E; X20); D) Plenty of bacilli evident in the cytoplasm of histiocytes forming globi and also lying free; BI - 6+ (FF stain, X100); E) Large number of lepra bacilli; BI - 6+ (AR stain, X100)

Out of 60 biopsy samples in the present study, 41 belonged to male patients and 19 to female patients. Leprae bacilli were identified in 42 patients (70%) on Fite-Faraco staining, while Fluorescent microscopy with A-R stain showed positivity for leprae bacilli in 48 cases (80%) (Table 1**).** In 18 cases, acid-fast bacilli (AFB) by Fite Faraco was not detected, and histopathologic findings correlated with the clinical picture to diagnose Hansen’s disease. These cases were clinically all diagnosed as paucibacillary. However, in the current study on fluorescent microscopy with A-R stain, bacilli could be detected in 6 out of 18 cases labeled negative on FF stain**.** FF stain detected bacilli in 66.6% of Indeterminate Leprosy (Figure 1), 50% of Tuberculoid type (Figure 2), 41.6% BT cases, (Figure 3,) 85.7% of BL cases (Figure 4), 100% of BB/MB (Figure 5) and LL cases (Figure 6), while A-R stain detected lepra bacilli in 50% of TL cases, 54.1% of BT cases and all 100% of IL, BL, BB, and LL cases. (Table 1) When bacteriological index was estimated on all tissue samples, the mean bacteriological index calculated by Fluorescent stain (2.48) was significantly higher than that by FF stain (2.18). It had a positive and strong correlation (Pearson ’r’ = 0.93) and was also statistically significant (*P*<0.00001). (Table 2) Out of total 32 cases categorized as Paucibacillary (BI < 2) on FF stain, 9 were re categorized into Multibacillary (BI > 2) on A-R Stain. (Table 3). 

**Table 1:** Comparison of positivity rates of FF stain and AR stain in Hansen’s Disease

Histopathology diagnosis	No. of Cases	FF Stain	A-R Stain
		Positive (%)	Positive (%)
Indeterminate Leprosy (IL)	3	02 (66.6)	3 (100)
Tuberculoid Leprosy (TL)	2	01 (50)	01 (50)
Borderline Tuberculoid Leprosy (BT)	24	10 (41.6)	13 (54.1)
Mid Borderline Leprosy (BB)	01	01 (100)	01 (100)
Borderline Lepromatous Leprosy (BL)	14	12 (85.7)	14 (100)
Lepromatous Leprosy (LL)	16	16 (100)	16 (100)
Total	60	42 (70)	48 (80)

**Table 2:** Correlation of Bacteriological Index (BI): FF stain and A-R Stain in Hansen’s Disease

Histopathology diagnosis	No. of cases	Mean BI		Pearson ‘r’	P-value
		FF Stain	A-R stain		
Indeterminate Leprosy (IL)	3	1.6	2	-	-
Tuberculoid Leprosy (TL)	2	1	1.5	-	-
Borderline Tuberculoid Leprosy (BT)	24	0.4	0.8	0.771	0.00001
Mid Borderline Leprosy (BB)	01	3	4	-	
Borderline Lepromatous Leprosy (BL)	14	3.5	3.7	0.855	0.000097
Lepromatous Leprosy (LL)	16	3.8	3.9	0.939	< 0.00001
Total	60	2.18	2.48	0.931	< 0.00001

**Table 3:** Comparison of bacteriological index in FF stain and AR stain on tissue section in Hansen’s Disease

Type of Leprosy	Pauci bacillary (A-R stain)	Multi bacillary (A-R stain)	Total
Pauci bacillary Leprosy - (FF stain)	23	09	32
Multi bacillary Leprosy - (FF Stain)	04	24	28
Total	27	33	60

**Table 4:** Comparison of Positivity of Lepra bacilli on FF and AR stain in Hansen’s Disease

S No	Studies: Literature Review	FF stain positivity (%)	AR stain positivity (%)
1	Nayak et al. (6) - 2003	25 (44.64)	39 (69.64
2	Adiga et al. (14) - 2016	19 (31.7)	26 (43.3)
3	Selfu Girma et al. (15) - 2018	87 (77)	88 (77.9)
4	Kalagarla S (17) – 2020 (n=40)	20 (50)	27 (67.5)
5	Present study - 2021 (n=60)	42 (70.0)	48 (80.0 )

## Discussion

Leprosy is the oldest disease known to mankind (11). Approximately 296,499 people live in areas where leprosy is a significant problem (12). There is an increasing demand for new techniques that are more accurate in identifying lepra bacilli and can provide clinicians with quick, accurate, and reliable results. The conventional method of diagnosing leprosy involves histopathological examination followed by the demonstration of acid-fast lepra bacilli, usually through the Fite-Faraco method. This histochemical stain uses Ziehl-Neelsen carbol fuchsin solution on a microscopic slide prepared with xylene-peanut oil to penetrate the mycolic acid-rich cell walls of *Mycobacterium leprae*. Because the leprosy bacillus is more easily decolorized than the tubercle bacillus, careful control is required during differentiation. As a result, 5% sulfuric acid is used as a decolorizer instead of an acid-alcohol solution. The auramine-rhodamine stain used for fluorescent microscopy also binds to mycolic acid. The acid-fast bacilli fluoresce red-orange, yellow, or reddish-yellow, whereas non–acid-fast bacilli do not fluoresce and appear pale yellow.

Using fluorescent microscopy with auramine-rhodamine stain has several advantages over the Fite-Faraco stain, facilitating better visualization of lepra bacilli, which appear as bright fluorescing rods against a dark background. This significantly reduces observer fatigue and screening time, leading to quicker identification, more accurate quantification of bacilli, and increased sensitivity (7).

Diagnosing leprosy in its early stage is clinically challenging, even though early detection offers the potential for definitive treatment. Fluorescent microscopy may be particularly helpful in diagnosing these early stages, in paucibacillary disease, and within the indeterminate spectrum of Hansen’s disease, which is often missed on histopathology due to the absence of a clear epithelioid or macrophage granuloma and the scarcity of acid-fast lepra bacilli (13). Previous studies have reported a higher bacillary index (BI) with fluorescent microscopy compared to the Fite-Faraco stain (Table 4). The present study also showed a higher positivity rate in detecting lepra bacilli with fluorescent staining compared to Fite-Faraco, aligning with various literature reports such as those by Nayak* et al.* (6), Jariwala* et al.* (14), Adiga* et al.* (15), Girma* et al.* (16), and Kalagarla S* et al.* (17). These authors have even re-categorized cases from paucibacillary to multibacillary, which has major therapeutic and prognostic implications (15).

In the present study, fluorescent microscopy proved more sensitive in detecting bacilli in 33% of overall negative cases. It was particularly useful in the borderline group, as more cases had undetected bacilli by the Fite-Faraco stain in this category. *M. leprae* is not cultivable in vitro. Alternative diagnostic methods for detecting acid-fast lepra bacilli include polymerase chain reaction (PCR), which has been used for challenging cases such as indeterminate and pure neuritic leprosy (18,19). In diagnostically difficult scenarios—especially in the tuberculoid spectrum and indeterminate leprosy with a very low bacillary load—serological markers like phenolic glycolipid-1 (PGL-1) for detecting *M. leprae* or immunohistochemistry with S-100 protein can serve as adjuncts to histopathology for demonstrating nerve damage and reaching a definitive diagnosis of Hansen’s disease (20). However, these methods involve more complex laboratory procedures and higher costs.

## Limitations

Fluorescent microscopes are expensive diagnostic modality and trained and experienced technical staff and is needed to identify the lepra bacilli.

## Conclusion

Screening of Auramine-rhodamine–stained sections requires significantly less time and is less taxing for the observer. It is a more sensitive modality, making fluorescent microscopy advantageous as a supplement to the conventional Fite-Faraco stain, especially in cases where the latter fails to detect leprae bacilli. The presence of bacilli and the assessment of bacillary load in tissue sections have a significant impact on treatment options. The higher pick-up rate of bacilli with fluorescent microscopy helps avoid undertreatment of leprosy by reducing false negatives, thereby decreasing treatment failure and relapse rates.

## Abbreviations

AFB: Acid Fast Bacilli

A-R Stain: Auramine Rhodamine stain

BI: Bacteriological Index

BL: Borderline Leprosy

BT: Borderline Tuberculoid Leprosy

BB: Mid borderline or Bordeline Borderline Leprosy

FF: Fite Faraco stain

FM: Fluorescent Microscopy

LL: Lepromatous Leprosy

PCR: Polymerase Chain Reaction

TL: Tuberculoid Leprosy
